# The Treatment of Leukoplakia by Radium

**Published:** 1916-11

**Authors:** Russell H. Boggs

**Affiliations:** Pittsburg, Pa., Roentgenologist, Allegheny General Hospital; Dermatologist and Roentgenologist, Pittsburg, Columbia and St. Francis Hospitals


### RADIUM DEPARTMENT

Under the Editorial Supervision of Dr. Edward Holman Skinner, of Kansas City Mo. Assisted by Dr. J. M. Martin, of Dallas, Texas.

Address communications to Dr. Skinner or Dr. Martin.

### "The Treatment of Leukoplakia by Radium."

BY RUSSELL H. BOGGS, M. D., PITTSBURG, PA., ROENTGENOLOGIST, ALLEGHENY GENERAL HOSPITAL; DERMATOLOGIST AND ROENTGENOLOGIST, PITTSBURG, COLUMBIA AND ST. FRANCIS HOSPITALS.

Radium has proved a very useful agent in the treatment of leukoplakia, which has been very resistant to most forms of treatment. It is a forerunner of epithelioma in many instances and should receive just as prompt and efficient treatment as precancerous lesions on the skin.

Leukoplakia is a disease which attacks the mucous membrane of the tongue, the inner surface of the cheeks, gums, lips, roof of the mouth and rarely the vagina and genitals. It is characterized by one or more whitish patches, often more or less thickened. The surface is smooth, roughened or somewhat papillomatous, and there may be an encircling hyperemia. The course is chronic and ulceration and malignant changes frequently occur. In some cases the lesions, after reaching a certain stage, remain stationary for a long time. The process is one of proliferation of the cells of the rete malpighii with cellular infiltration in the papillary layer of the corium. When examined closely, these lesions are found to be made of a hyperkeratinized epithelium, being covered by adherent and more or less dense pellicle, removable only by artificial means and closely applied to the inferior stratum of the mucous.

Views on buccal leukoplakia have given birth to a series of hypotheses that have been enunciated one after the other during the last few years. It is generally conceded that there are two forms of leukoplakia; first, the true idiopathic, the cause of which is unknown and which alone leads on to an epithelioma in the form of the so-called smoker's cancer; and the false, which is a form of tertiary syphilis, always more or less sclero-gummatous, and, according to some authorities, is never followed by cancer. Some authorities, to the contrary, state that there are two principal factors in the production of cancer of the mouth and in most instances they are both present, namely, syphilis and the use of tobacco. Without denying the great frequency of tertiary syphilis of the mouth and its possible transformation into cancer, there is no question of the existence of the non-syphilitic leukoplakia, chronic from the onset, followed by epitheliomatous degeneration. It makes its appearance almost exclusively in men between the ages of forty and sixty. It is different from the syphilitic lesion, and as a matter of fact should be treated as a superficial epithelioma. Syphilitic leukoplakia is accompanied by induration, fissures and later by scarring and white fibrous tracts. The diagnosis may be confirmed by a Wassermann. I feel sure that many of the syphilitic lesions of the mouth later become cancerous, but this type of malignancy does not respond readily to any form of treatment. It has been shown in the past that the treatment of true leukoplakia has not been satisfactory from the irritating methods of cauterization. When first seen the lesions are very superficial and it is not necessary to use a method which will destroy tissue at a depth more than one or two millimeters. On account of the successful results which I have obtained in the treatment of epithelioma with radium and on account of its easy application to the mucous membrane of the mouth, I decided to employ radium in my first case, almost three years ago. The results were so satisfactory that I have been treating all cases of true idiopathic leukoplakia as well as a few cases of syphilitic leukoplakia ever since. The latter have not responded nearly so well, but accomplishes more even in the leutic type than any other local form of treatment we possess today, because I believe in many instances we have an epithelioma situated in syphilitic tissue and will not respond to one form of treatment alone. In true leukoplakia we must abstain from giving iodides or compounds of iodine, the effects of which are deplorable. Many of the cases have had such treatment before they were referred, which only aggravated the condition. It is necessary to make an early diagnosis since all irritating treatment is contra-indicated. It is a waste of time to use gargles and inert medication.

In the treatment of leukoplakia the best results are obtained by the rays of low penetration, as it is not necessary to have a reaction deeper than one or two millimeters. I have been using one-half a millimeter of silver, several layers of gauze and a finger cot as a filter. This allows a large amount of the Beta rays to be used in the treatment. Whether this is absolutely essential

I am unable to state, but, by using this amount of filtration, the time of exposure can be considerably reduced. Using a tube containing 25 milligrams of the element, one to two hours is usually sufficient for each lesion depending upon its stage and extent. On the tongue, on account of the papillae, it requires a slightly larger dose than on the buccal membranes, but I always attempt to clear up the lesion with the smallest amount of irritation possible. This is contrary to my usual procedure in epithelioma of the skin or carcinoma of the cervix or uterus, where intensive radiation is given as quickly as possible. This coincides with many observers and is the principal reason why results are so unsatisfactory from irritating caustics.

In this connection let me cite a case to illustrate the usual course of many of the leukoplakias. Mr. M., age 50, had a leukoplakia on the left inner side of the cheek. It was treated by caustics, first by his dentist, again by his family physician, and a year later was removed surgically and the pathological report was epithelioma. It recurred, and a second operation was performed. Three months after his last operation he was referred for radiotherapy with a large infiltrated growth in his mouth, which was broken down and very offensive, and he was scarcely able to open his mouth. The glands were involved and he was suffering a great deal of pain. Radiotherapy relieved the pain, killed the offensive odor and checked the disease, but he died eight months later. These are the cases that should have early radium therapy instead of inert irritating treatment. We all have witnessed precancerous lesions in the mouth following similar sad experiences, seeing the disease progress under inefficient treatment. Epithelioma in the mouth is always a serious condition if the disease is advanced and the results produced by radium will, in the future, bring patients early so they can be treated in the precancerous stage. Leukoplakia in the past has been considered a trivial lesion and from the description in text-books it is not any wonder that it is so considered by many.

In conclusion, radium has proven to be an efficient form of treatment. Leukoplakia is frequently a forerunner of epithelioma and should always be seriously considered.

Even when leukoplakia has become epitheliomatous, radium is the best form of treatment we possess today and some remarkable results have been obtained, but a guarded prognosis must always be given if the disease is advanced and there is glandular involvement.

